# Insanity and Its Kinships

**Published:** 1894-03-10

**Authors:** 


### The Hospital

An Institutional Journal of

The Medical Sciences and Hospital Administration, with

Vol. XV.—No. 389.] AN EXTRA NURSING SUPPLEMENT. [March 10, 1894.

### Insanity and its Kinships.

The dry light of reason is of all lights the most illuminating to the human mind. It is a proposition equally true that the genial warmth of sympathy is a great aid towards the proper use of the dry light. Insanity in the past has been a terrible human ill. It begins now to be robbed of its terrors, thanks to the twin guidance of reason and sympathy. Not yet, however, does the great world know all about insanity that science knows; and not yet do law and common sense act with full justice towards those who are so unhappy as to be insane. More knowledge is needed on the part of statesman, lawyers, and common people; and much prejudice, which appears to be almost inveterate, must by some means be removed before the lunatic can obtain entire fair play at the hands of his sane fellow man.

Dr. T. S. Clouston, medical superintendent of the Morningside Asylum at Edinburgh, whose annual statement has just been published, is a man whose every word, at any rate in Edinburgh, where he is known, is weighed and treasured by those who are most competent to value the utterances of men. This position of reasonable authority Dr. Clouston has gained by more than a quarter of a century's excellent scientific and conscientious work in the field of lunacy. What thoughtful Edinburgh values Great Britain cannot afford to despise. We desire to claim for Dr. Clouston's conclusions that high authority he has so decidedly established for himself, and which it would be so advantageous to the whole British public to recognise.

The "kinships of insanity"! It is almost a novel idea to some persons that insanity should be nearly related to other bodily ills, and that among such ills it should not have one or two relations merely, but a numerous band. What are those diseases which are related to insanity by the closest ties of consanguinity, the ties of actual brotherhood? "By its own nature, and by heredity," says Dr. Clouston, "lunacy is own brother to epilepsy, convulsions, dipsomania, paralysis, idiocy, and St. Vitus' Dance." To certain other forms of bodily illness it has a kinship hardly less remote. It is "first cousin," to quote Dr. Clouston again, "to neuralgia asthma, diabetes, and nervous exhaustion!" These are words which have a wide range of significance. If they expand the conception of insanity, they also show how many ills that at present are looked upon as merely bodily may nevertheless be associated with all kinds of mental and moral phenomena, for which the person in whom any one of these bodily diseases manifests itself may be in no wise responsible. In other words, if the epileptic and even the dipsomaniac be "own brother" to the insane, is it right and just that these should be treated as if they were in possession of an unimpaired sanity? If the victim of neuralgia, asthma, or diabetes be "first cousin" to the lunatic, it is obvious that in every relation of life this fact must be taken into account. But how many people outside the medical profession—nay, even outside that limited number of the members of the medical profession who scientifically study the phenomena of alienism—know anything whatever about these facts?

The conclusions set forth by Dr. Clouston are among those many discoveries and conclusions of science which have entirely failed to find their way into the regions of common knowledge. They show to the scientific, if not to the untutored man, that scientific civilisation is still in its infancy. But the civilisation of the future must be scientific, or if it be not there will be no future at all for the races that now lead; that is to say, the knowledge of all kinds which science masters must be constantly added to the common stock of knowledge and to the guiding factors of common mankind. It must be incorporated with our laws, it must correct and enlarge our opinions and judgments, it must determine our daily conduct. This is only the same as saying that scientific knowledge is to be utilised by man in the same way as he has utilised every other available kind of knowledge and experience. But hitherto science has been the property of a class, and the unscientific among us, which, unhappily, include most of our legislators and administrators of the law, as well as the more crude and ignorant masses of mankind, have regarded science as a child regards the tricks of the conjurer, as something to be amazed at, but not to be mastered and used.

Those persons who help to make public opinion, those also who make and administer our laws, and those who devote their lives to the study and teaching of morals and religion, are bound by the most sacred obligations to study such questions as the causes and consanguinities of insanity, and to make themselves competent judges and authorities therein. If they do not, how are they to ensure justice between man and man, and especially justice between the strong man who is entirely sane, and the helpless or partially helpless man who is only partly or occasionally sane? Even the presiding genius of the prize ring feels it his bounden duty to see fair play. How much more should lawyers and judges provide themselves with that full knowledge of real and demonstrated facts which will enable them to do real and solid justice between all classes of the enforced gladiators who crowd the arena of common life. Is it true that the dipsomaniac and the epileptic are own brothers to the insane? Is it true than lunacy, asthma, diabetes, and neuralgia are blood relations and first cousins? Many, even among medical men, may question the full truth of this latter proposition. But Dr. Clouston is not a man to commit himself to conclusions which he has not verified. The man of least knowledge in our profession is well aware that those diseases which are said to be first cousins to insanity have their roots and origins in the nervous system just as insanity has. Asthma is a disease of the nervous system however we may trace it a step further and find elsewhere the poison to which that system yields before it produces the phenomena of asthma. Similarly diabetes is a disease of the nervous system, even though it should ultimately be proved that the pancreas plays a part in the inception of the disease which until recent years had not been so much as suspected. Notwithstanding any plausible objections which may be urged against Dr. Clouston's wide and far-reaching range of alienistic vision, the practical physician, whether alienist or not, who has systematically used the habit of deep and philosophic observation cannot but feel that here is a profound view into the inner truth of physiological facts, and that it behoves all right-minded men to consider what is set before them, and if they find it on investigation to be demonstrably true to incorporate it with their inner convictions, and to use it honestly in their working lives.

"Justice to the insane" is one of the noblest mottoes which a scientific and humane civilisation can inscribe upon its banner. To the clear-seeing physician it pierces even deeper into the tenderest human sympathies than the plea of "pity for the sick." Justice to the unhappy human brother who no longer has the mental capacity to compel justice for himself, or even to state his own claim. In asking for this justice few readers are aware how nearly they themselves or their families may be concerned in the plea. "Nothing is more common," says Dr. Clouston, "than the boast that our family at least is quite free from insanity." A very futile and ignorant boast this almost invariably is. "It might make the world," he continues, "more pitiful and tolerant, and a little humbler if the plain facts were fully realised. There are few families indeed who are free from either mental disease or those brothers and first cousins of that disease which may, by marriage, be transmuted into insanity in the next generation." Self-interest should make us all light-seekers on the subject of the causes and kinships of insanity: a nobler motive, the offspring of scientific intelligence and justice, will carry us quickly to the desired knowledge, and make us lay firm hold upon it for immediate service, as the old-time warrior grasped his trusty and wrong-righting sword.

